# Whole Blood Metabolomics in Aging Research

**DOI:** 10.3390/ijms22010175

**Published:** 2020-12-26

**Authors:** Hiroshi Kondoh, Masahiro Kameda, Mitsuhiro Yanagida

**Affiliations:** 1 Geriatric Unit, Graduate School of Medicine, Kyoto University, Kyoto 606-8507, Japan; mkameda@kuhp.kyoto-u.ac.jp; 2 G0 Cell Unit, Okinawa Institute of Science and Technology Graduate University (OIST), Okinawa 904-0495, Japan; myanagid@gmail.com

**Keywords:** aging, metabolites, frailty, fasting, antioxidant, metabolomics, whole blood

## Abstract

Diversity is observed in the wave of global aging because it is a complex biological process exhibiting individual variability. To assess aging physiologically, markers for biological aging are required in addition to the calendar age. From a metabolic perspective, the aging hypothesis includes the mitochondrial hypothesis and the calorie restriction (CR) hypothesis. In experimental models, several compounds or metabolites exert similar lifespan-extending effects, like CR. However, little is known about whether these metabolic modulations are applicable to human longevity, as human aging is greatly affected by a variety of factors, including lifestyle, genetic or epigenetic factors, exposure to stress, diet, and social environment. A comprehensive analysis of the human blood metabolome captures complex changes with individual differences. Moreover, a non-targeted analysis of the whole blood metabolome discloses unexpected aspects of human biology. By using such approaches, markers for aging or aging-relevant conditions were identified. This information should prove valuable for future diagnosis or clinical interventions in diseases relevant to aging.

## 1. Diversity of Human Aging Exposes the Limits of Calendar Age

Currently, Japan is the only country in the world with a societal aging rate (% of population over 65 years old) exceeding 30% (global average = 12.38%) [1,2]. However, a wave of global aging is steadily approaching, and the global average is expected to reach 23.4% by around 2050 [1]. Indeed, many European countries, China, South Korea, Thailand, Singapore, Iran, Chile, Canada, and others will reach aging rates of more than 25%, while Japan is expected to exceed 42.5% by that time [1].

One of the major causes of this global aging is improved health of the elderly especially due to medical progress. However, the causes of “aging” change through time. As one leading cause of death is overcome, another takes its place. For example, immediately after World War II, antibiotics helped to eliminate many infectious diseases, thereby extending life expectancy, while in the 1960s, cerebral hemorrhage was a major reason that elderly people became bedridden [3,4]. Now, we observe greater “diversity of aging” in countries with a high proportion of elderly people, including Japan. As the number of healthy elderly people increases, the number of bedridden patients and people who need long-term nursing care increases as well.

Because “diversity of aging” reflects individual variability, clinical symptoms among the elderly are also diverse. For example, atherosclerosis is a well-known disease that accompanies aging. Endothelial thickening of the carotid artery and increased vascular pulse wave velocity are indicators of arteriosclerosis, which predicts vascular age [5]. Average values for such parameters in the elderly are worse than those of young people. Careful observation reveals that these indicators show uniformly low values among the young, while values measured in the elderly range largely from low to high [5]. It is true that there are some older people who have better indicator values than younger people. These findings demonstrate that “aging” is multi-faceted and complex, and that variability is characteristic of aging.

Calendar age has conventionally been used as an indicator of human aging. According to calendar age, the elderly are classified into four groups: Early elderly (65 to 74 years old), late elderly (over 75 years old), super-elderly (over 90 years old), and centenarians, people over 100 years of age. This definition, based on calendar age, was proposed by the United Nations in 1956. However, as we are now facing global aging of the population and diversification of the elderly, redefinition of “elderly” itself is becoming necessary (proposal of the Japanese Geriatrics Society in 2017).

## 2. The Aging Hypothesis Relative to Metabolic Profiles

**Figure 1:**
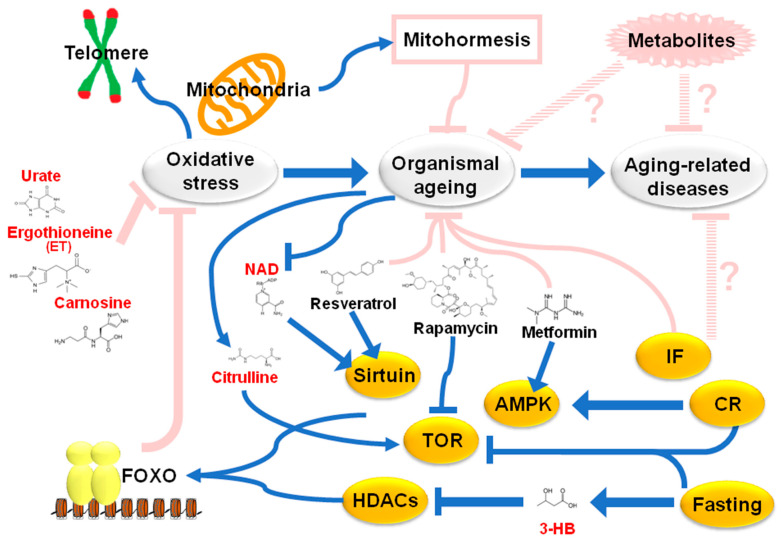
The aging hypothesis relative to metabolism. Earlier, “the oxidative damage hypothesis” or “mitochondrial aging hypothesis” was proposed by Harman, as oxidative stress reduces organismal lifespan. Calorie restriction (CR) or intermittent fasting (IF) effectively extends lifespans of experimental models. During CR, several signal modules, including sirtuin, Tor kinase, and AMPK, are activated or inactivated. Consistently, chemicals or compounds mimicking CR conditions increase longevity (resveratrol for sirtuin, rapamycin for Tor kinase, and metformin for AMPK). One of the targets of these signals is FOXO transcriptional factor, which activates radical scavengers. However, recent findings on mitohormisis challenge the mitochondrial aging hypothesis. Moreover, little is known about the effect of CR or fasting on human longevity. Some of the metabolites described in this review (red color) are known to interact with the signal molecules or to serve as antioxidants. HDACs: Histone deacetylase.

In addition to calendar age, the importance of “biological age” has also been proposed, mainly based on findings in basic aging research. For example, “telomeres” are known as “aging clocks” in replicative senescence. Other attempts to explain metabolic aspects of “aging” include the “oxidative stress hypothesis” and the “calorie restriction hypothesis” (Figure 1).

The former is one of the oldest aging hypotheses, proposed by Harman (1956) [6]. During his search for chemical inducers of DNA mutations, he became aware of the highly reactive properties of “free radicals.” Damage to large molecules caused by free radicals is known as “oxidative stress.” Telomeric DNA is also impaired by oxidative stress [7]. Oxidative stress was proposed as a cause of aging by Harman and more recently as a factor in chronic inflammation. Indeed, lifespan extension in experimental models was promoted by ectopic expression of “radical scavengers”, such as catalase and super oxide dismutase (SOD) [8,9]. In addition, over 90% of oxidative stress has been attributed to mitochondria [10]. The “Mitochondrial Aging Hypothesis” proposed by Harman (1972) suggested that oxidative stress greatly increases biological age [11]. Consistently, the genetic manipulation to extend the lifespan is known to be accompanied with the increase of antioxidants; for example, glutathione has been elevated in super Arf/p53 mice, a longevity model [12].

When calorie intake is reduced by 20–30%, lifespan is extended by 20% or more in many model organisms, such as mice, flies, fish, spiders, etc. [13,14]. Calorie restriction (CR) activates and modulates several signal responses, such as sirtuin, AMPK kinase, Tor kinase, and FOXO transcription factor [15,16,17,18,19,20]. Targets of FOXO transcription factor include a group of radical scavenger genes. Consistently, during CR, reduction of oxidative stress is observed, indicating the role of oxidative stress in the “calorie restriction hypothesis” [21,22]. A recent report suggested that the modulation of FOXO via peptide intervention induces elimination of senescent cells in vivo, followed by healthy lifespan [23].

Interestingly, metabolites modulating these signaling pathways are effective both for extension of organismal lifespan in experimental models and for treatment of human diseases of aging: Resveratrol (activator of sirtuins) against obesity, rapamycin (inhibitor for Tor kinase) as an anti-cancer drug or immunosuppressor, and metformin (activator for AMPK) in diabetic therapy [17,24,25,26]. NAD+ also activates sirtuins, while lifespan is extended by adipose-tissue-specific expression of Nampt, which mediates NAD+ biosynthesis [27]. Moreover, nicotinamide mononucleotide (NMN), a precursor of NAD+, alleviates age-related functional decline in rodents [28]. However, as ectopic expression of FOXO compromises metabolic regulation in mice, it is not clear whether modulation of FOXO would be efficacious in clinical applications [29,30].

Moreover, the mitochondrial aging hypothesis has been challenged since about 2005. Recent genetic studies suggest that lifespan in *Caenorhabditis* and *Drosophila* are extended by partial inactivation of mitochondrial functions, such as mitochondrial SOD, mitochondrial complex protein, and mitochondrial ribosomal proteins [31,32,33]. Moreover, it was observed that very limited but defined overload of “mitochondrial oxidative stress” (“mitohormesis” [34]) extended lifespan in nematodes.

These conflicting findings on oxidative stress and longevity cannot be overlooked. In human clinical trials, supplementation with the antioxidants, beta-carotene, vitamin A, or vitamin E reduced mortality [35,36]. Regarding the “calorie restriction hypothesis”, long-term clinical studies by CR intervention in humans are very difficult, except for obesity or diabetes, as some epidemiological studies suggest that slightly overweight people live longer [37]. Compared to that in model organisms, human aging research may require another approach, in part due to its variability and complexity.

Other than CR, supplementations of several metabolites are under investigation, for example NMN [38], as intervention approach against human aging. The combination of exercise and supplementation for branched-chain amino acids strengthens lower limb muscles in physical frailty [39]. Thus, metabolites could be a promising strategy for intervention against aging.

## 3. Metabolomic Approach for Human Whole Blood

Metabolites are small organic compounds, generated by the metabolic activity of living organisms, from bacteria to humans. The metabolome comprises numerous metabolites that may be quantified with liquid chromatography–mass spectrometer (LC-MS). Analysis of the metabolome (metabolomics) is developing as a powerful and useful tool, not only to search for diagnostic metabolites or biomarkers, but also to provide valuable information about metabolic profiles of tissues, cells, media, urine, and blood, in health and disease.

Among other things, human blood is convenient and useful to analyze, because it reflects in vivo physiological states influenced by genetics, epigenetics, health, and lifestyle [40,41]. Thus, metabolomics of human blood can provide comprehensive information on metabolic mechanisms of physiological responses, health, aging, and diseases, and of biological effects of drugs, nutrients, and environmental stressors [42,43,44,45,46].

Blood comprises cellular and non-cellular components: Red blood cells (RBCs), white blood cells (WBCs), platelets, and plasma. Many studies on blood have been conducted on plasma or serum, the non-cellular component [47,48]. One reason is the difficulty in handling cell-derived metabolites so as to promote their stability [49].

We pursued and established our own method for analyzing whole blood, plasma, and RBCs. The whole blood metabolome includes about 130 metabolites, comprising over 14 subgroups (nucleotides, nucleosides, nucleobases, vitamins and coenzymes, nucleotide-sugar derivatives, sugar phosphates, sugar derivatives, choline and ethanolamine derivatives, organic acids, anti-oxidants, amino acids, methylated compounds, acetylated compounds, carnitines, etc.) [50,51,52,53]. These reflect cellular metabolism (energy production, DNA and RNA synthesis, lipid metabolism, mitochondrial respiration, redox homeostasis, protein synthesis, and so on). Hence, levels of these compounds are influenced or regulated by various activities of tissues, activation or inactivation, secretion or absorption, regeneration or degradation, contraction or relaxation, digestion or condensation, accumulation, or excretion, etc.

Among the ~130 metabolites assessed by whole blood metabolomics, 50–60 are enriched in RBCs [52,53]. Individual differences in each metabolite were also evaluated [53]. Based on the coefficient of variation (CV: SD divided by the mean), metabolites are classified into two subgroups: Those that are less variable (CV < 0.4) and others with large variability (CV > 0.4 or higher). The former comprise many essential metabolites (ATP, glutathione, phospho-sugars, etc), while the latter include dietary compounds such as caffeine, carnosine, ergothioneine, 4-aminobenzoate, and others [53]. Thus, our metabolomic approach to whole blood comprehensively captures complex changes with individual differences, suitable for human aging research. Indeed, whole blood metabolomics have revealed metabolites that serve as biomarkers relevant to aging, fasting, and frailty [53,54,55,56].

## 4. Whole Blood Metabolites for Aging and Fasting Markers

### 4.1. Metabolites for Aging

While targeted metabolomic analysis has identified aging-relevant compounds [46,47,57,58], a comparative, non-targeted analysis of the whole blood metabolome was carried out to compare healthy young and elderly people [53]. Among the 126 metabolites identified, there were statistically significant differences in 14 metabolites (11%) between young people (29 ± 4 years old) and elderly (81 ± 7 years old), which can be regarded as aging markers. Six of them are RBC-enriched, suggesting that whole blood metabolomics is important for human aging research.

Nine of the 14 metabolites decreased in the elderly, while five of them increased (Table 1). The former include 1,5-anhydroglucitol (1,5-AG), acetyl-carnosine, carnosine, ophthalmic acid (OA), leucine, isoleucine, nicotinamide adenine dinucleotide (NAD^+^), nicotinamide adenine dinucleotide phosphate (NADP^+^), and UDP-acetyl-glucosamine, while the latter comprise citrulline, pantothenate, dimethyl-guanosine, *N*-acetyl-arginine, and N6-acetyl-lysine.

The 1-deoxy form of glucose, 1,5-AG, is derived from many foods. Circulating 1,5-AG is excreted from renal glomeruli, but reabsorbed through renal proximal tubules. As glucose is a competitive inhibitor of reabsorption of 1,5-AG, diabetic patients with high blood glucose levels show lower 1,5-AG levels. Thus, 1,5-AG is a known diabetes marker. However, levels of HbA1c and serum glucose, other diabetic parameters, are normal in both healthy young and elderly in this study; hence, it is possible that re-absorption of 1,5-AG in the kidneys is reduced with aging even in healthy people [53]. It is noteworthy that 1,5-AG also plays a role in oxidative defense. Carnosine and acetyl-carnosine are dipeptides with antioxidant properties, which are abundant in muscles and neurons [59,60].

OA is a tripeptide analog of glutathione, and l-γ-glutamyl-l-α-aminobutyrylglycine is synthesized by some of the same enzymes (glutathione synthase) utilized for glutathione production. Because glutathione is one of the most abundant antioxidative compounds in cells, OA is known as an oxidative stress marker [61]. NAD^+^ and NADP^+^ are coenzymes involved in various redox reactions. Leucine and isoleucine are essential amino acids involved in maintenance of skeletal muscle [62]. Recent blood metabolomics study consistently identified reduction of essential amino acids in elderlies [63]. UDP-acetyl-glucosamine is a substrate for glycosyl-based modifications and is involved in synthesis of proteoglycans and glycolipids [64,65].

Among metabolites that increase in the elderly, citrulline is synthesized in the urea cycle, and is involved in excretion of nitrogen. Dimethyl-guanosine and *N*-acetyl-arginine are also related to nitrogen metabolism [66,67]. These results suggest that urinary discharge of nitrogen-related metabolites is reduced in the elderly. Pantothenate is a precursor of coenzyme CoA, involved in the TCA cycle and β-oxidation.

Correlation analysis among these 14 aging compounds suggests strong correlations among metabolites with declining values in elderly, and also among those that increase in the elderly. However, interestingly, there was no negative (reverse) correlation between these two subgroups, indicating at least two distinct subgroups for aging metabolites.

Collectively, these 14 metabolites include antioxidants, nitrogen, and muscle- or kidney-related metabolites, reflecting a decline or impairment in specific physiological functions in the elderly. Such functional declines have been assumed in aging, but have rarely been discussed as conventional indicators. Thus, whole blood metabolomics identified both undesirable accumulations and deficiencies of metabolites in aging.

### 4.2. Fasting Responses; Energy Substitution

As mentioned above, the positive impact of CR on organismal longevity has been well established in experimental models. Moreover, recent reports suggest that intermittent fasting, a cycle of fasting and feeding, enables *C. elegans* to live about 50% longer than conspecifics on a normal diet [68]. Thus, CR and intermittent fasting have overlapping roles in prolongation of lifespan. However, verification of the “calorie restriction hypothesis” in healthy, non-obese humans is rather difficult.

While little is known about the link between fasting and aging in humans, it is well known that humans can withstand 30–40 days of fasting if dehydration is avoided [55], in sharp contrast to the vulnerability of mice to hunger (average survival of only several days of starvation). For example, Gandhi experienced hunger strikes of up to 21 days, 14 times or more in his lifetime as a form of political protest [69].

Historically, research on fasting physiology has focused particularly on energy substitution [70,71,72,73]. Glucose normally constitutes the major fuel source under non-fasting conditions, but during fasting, glycogen stores are rapidly exhausted in an effort to maintain minimum glucose levels in the blood. After depletion of glycogen storage, in addition to gluconeogenesis, fasting forces the human body to utilize various non-carbohydrate metabolites, such as lipids and branched-chain amino acids (BCAAs) as energy sources [70,73]. Hormonal changes stimulate lipolysis in white adipose tissue (WAT) and liver. First, 3-hydroxybutyrate (3-HB) increases 30 to 60-fold and is converted into acetyl-CoA in the brain or other tissues as an alternative energy source [70]. Second, elevated acylcarnitines during fasting are essential for lipid transport into mitochondria [74]. Third, increased concentrations of branched-chain amino acids (BCAAs), mainly released from muscle, are also utilized in the mitochondrial TCA cycle or in liver lipogenesis [75]. Thus, elevation of butyrates, BCAAs, and acylcarnitines in circulating blood (quantified by targeted metabolomics or other techniques) serve as indicators of fasting.

Additionally, non-targeted, comprehensive analysis of blood metabolites during prolonged fasting was performed to gain insights into its health effects [54]. Therefore, we conducted an exhaustive analysis of nearly 130 metabolites from blood of four healthy young people for 58 h of starvation. As a result, this study revealed that more than one-third of metabolites increased, indicating much greater metabolic activation during fasting than expected. Among 44 increased metabolites, well-known fasting markers figure prominently (ketone bodies, carnitines, and BCAAs), consistent with previous findings [70,73,74].

### 4.3. Fasting Responses and Novel Fasting Markers

**Figure 2:**
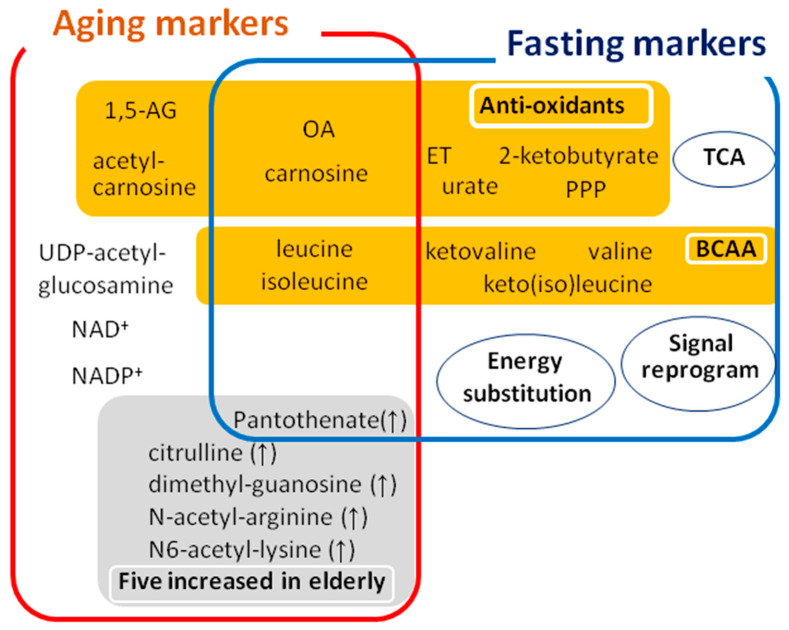
Whole blood metabolomics detect markers for aging and fasting. Overlapping, but distinct markers for aging and fasting are identified by non-targeted, comprehensive metabolomic analysis of human whole blood. Various antioxidative metabolites are included in the list of aging and fasting markers. In addition to known energy substitutions, unexpected aspects of fasting (antioxidants, mitochondrial activation, and signal reprogramming) were revealed. In response to 58-h fasting, 44 human metabolites increased, revealing remarkable metabolic activation. Metabolites increased during fasting did not overlap with the compounds increased in elderly, except pantothenate. Please notice that four metabolites decreased in elderly (OA, carnosine, leucine, and isoleucine) were increased during fasting, indicating the possible anti-aging effect of fasting. BCAA; branched chain amino acid, ET; ergothioneine, 1,5-AG; 1,5-anhydroglucitol (1,5-AG), OA; ophthalmic acid, PPP; pentose phosphate pathway.

In addition, several novel aspects of fasting were discovered involving (1) TCA cycle metabolites, (2) antioxidants, and (3) signaling molecules (Figure 2). First, an increase in TCA cycle metabolites reflects activation of mitochondrial function throughout the body during starvation, since red blood cells are not equipped with mitochondria. Increases of well-established fasting metabolites, ketone bodies, carnitines, and BCAAs, also support mitochondrial activity during fasting. Second, an increase in antioxidant compounds was also observed, in addition to urate, xanthine, carnosine, OA, and ergothioneine (ET). Urate is one of the most abundant antioxidants in blood [76], the precursor of which is xanthine. ET is abundant in mushrooms and fungi. In yeast, ET also increases in a low-glucose environment [77]. Additionally, four metabolites (6-phosphogluconate, glucose-6-phosphate, pentose phosphate, and sedoheptulose-7-phosphate) generated via the pentose phosphate pathway (PPP) increased in plasma, but not in RBCs during fasting. Activation of the PPP produces NADPH, which is essential for redox control [78]. As sugar phosphate compounds in blood are enriched in RBCs, PPP metabolite increases only in plasma suggest that responses in tissues are largely responsible for these altered profiles during fasting. Collectively, one of the most significant metabolic changes during starvation is antioxidant enhancement.

Third, whole blood metabolomics identified purines and pyrimidines (GTP, CTP, ADP, IMP, cytidine, and adenine) and some signal-modulating metabolites (3-hydroxybutyrate and 2-oxoglutarate) as fasting markers [54]. It is conceivable that fasting provokes global remodeling of transcriptional networks to adapt to metabolic changes. Increased purines and pyrimidines may support anabolic metabolism for RNA and protein synthesis; 3-hydroxybutyrate (3-HB), a major energy substitute during fasting, is also known as a histone deacetylase inhibitor [79], while 2-oxoglutarate activates 2-oxoglutarate oxygenase, functioning in demethylation of histones and nucleic acids, and destabilization of transcriptional factors [80]. Fasting may genetically or epigenetically modify transcriptional networks via such metabolites. [79,80].

Interestingly, four metabolites that increase during fasting (carnosine, OA, leucine, and isoleucine) overlap with the decreased metabolites in elderly (Table 1 and Figure 2). It is possible that fasting may exert anti-aging or rejuvenile effect through upregulation of these aging metabolites.

## 5. Whole Blood Metabolome for Diseases of Aging

### 5.1. Metabolomic Study of Frailty

Most aging-related diseases are increasing in our globally aging society, including lifestyle diseases (hypertension, diabetes, obesity, osteoporosis, atherosclerosis, etc.), dementia, cancer, and others. Among other factors, the severity of frailty is most closely correlated with health risks of advanced age [81]. Frailty is a vulnerability to stressors, due to the declining physiological capacity of organs as a result of aging [82,83]. Because it results from age-related deterioration of multiple organ systems, frailty displays complex features, including cognitive dysfunction, hypomobility, and impaired daily activity.

Frailty is currently defined by three major diagnostic tools [84]. The Fried Cardiovascular Health Study Index (CHS) is useful to detect physical frailty [82], while the Rockwood Frailty Index covers its multimorbidity [85]. In contrast, the Edmonton Frailty Scale (EFS) or Tilburg Frailty Indicator, efficiently evaluates both physical and psychosocial aspects of frailty [86,87].

Four recent studies reported the metabolomic analysis of blood from frail elderly subjects [88,89,90,91]. However, these reports drew divergent, non-overlapping conclusions, stemming from different experimental designs. For example, the former two reports applied the Fried CHS index as a diagnostic tool, which lacks cognitive assessment, while the latter two utilized the Rockwood Frailty index. Since these studies were based on serum samples, we performed whole blood metabolomics for frailty. Our study was designed to cover multiple domains of frailty, by applying the EFS, the Japanese version of the Montreal Cognitive Assessment (MoCA-J) [92,93] and the Timed Up & Go Test (TUG) [94] as diagnostic tools [86], because the EFS is recognized as a valid and reliable measurement tool for identification of frailty [95] and is widely recommended in clinical guidelines [84].

**Figure 3:**
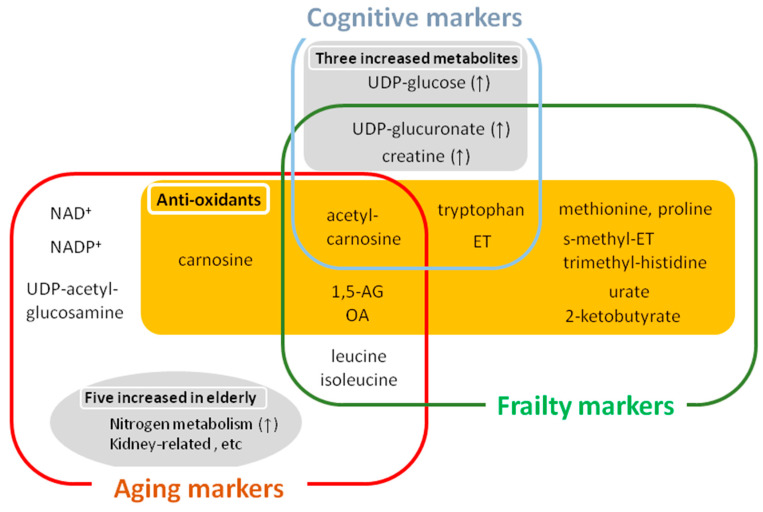
Metabolites for aging, frailty, and cognitive impairment. Various antioxidative metabolites are included in the list of aging, frailty, and cognitive markers. Among fifteen frailty markers, eleven anti-oxidative metabolites are decreased, indicating the involvement of antioxidative defense in the pathogenesis of frailty. Among fourteen aging markers, five metabolites (acetyl-carnosine, 1,5-AG, OA, leucine, and isoleucine) overlapped with frailty markers. Among six cognitive markers, five compounds (acetyl-carnosine, 1,5-AG, OA, UDP-glucuronate and creatine) overlapped with frailty markers, reflecting the cognitive aspects of frailty. Thus, cognitive markers are much involved in frailty markers, while one compound (acetyl-carnosine) are listed as overlapping both in aging and cognitive markers. Collectively, overlapping, but distinct markers for aging, frailty, and cognition are identified by non-targeted, whole blood metabolomics.

Using the EFS as a guide, our study identified 15 blood metabolites involved in antioxidation, cognition, and mobility as frailty markers (Figure 3) [56], while studies based on the Fried CHS reported blood metabolites related mainly to physical or sarcopenic frailty [89].

### 5.2. Frailty Markers for Anti-Oxidation, Cognition, and Mobility

First, among 15 frailty markers, seven compounds that decreased in frailty are associated with antioxidative defense: Acetyl-carnosine, ergothioneine (ET), S-methyl-ET, trimethyl-histidine, OA, 2-ketobutyrate, and urate (Figure 3). Trimethyl-histidine and S-methyl-ET are involved in ET synthesis (20); 2-ketobutyrate is a precursor of OA. Thus, the ergothioneine and OA pathways are greatly affected in frailty. Second, we observed that five amino acids (methionine, proline, tryptophan, isoleucine, and leucine) decreased significantly in frail subjects. Among these five amino acids, methionine, proline, and tryptophan, have been reported as radical scavengers in vitro [96,97,98,99]. Thus, our whole blood metabolome for frailty revealed antioxidant enrichment in cellular components. Consistently, recent findings of longitudinal studies support our notion that diminished antioxidative defenses are heavily involved in pathogenesis of frailty [100]. It is noteworthy that most cognitive markers and some hypomobility markers overlap with frailty markers, supporting the notion that frailty is an integrated spectrum of age-related disorders (Figure 3). On the other hand, only acetyl-carnosine overlapped in aging and cognitive markers, indicating the gap between physiological and pathological aging in cognition.

## 6. Summary

Interestingly, metabolites affected in frailty largely overlap with metabolites that decrease during aging (acetyl-carnosine, OA, 1,5-AG, isoleucine, and leucine) and compounds that increase during fasting (2-ketobutyrate, OA, isoleucine, leucine, urate and ET), indicating an intriguing metabolic link between frailty and human aging. Various antioxidative metabolites are conspicuously included among frailty and aging markers, suggesting that one of the key stressors to which frail elderly are vulnerable is oxidative stress. In this context, increased oxidative defense during fasting may effectively moderate aging or aging-relevant disorders.

Diversity of aging has a significant impact not only on clinical and basic research, but also on national economies, and facilitates structural changes in entire societies. The answer to the fundamental question, “What is aging?” is not yet fully known. Accumulating evidence suggests that defining “aging” only on the basis of chronological age does not explain the entire situation. Interestingly, some of the metabolites described in this review are known to interact with the signal molecules (Figure 1). NAD^+^, decreased in elderly, activates Sirtuin, while citrulline, accumulated in elderly, stimulates TOR kinase; 3-HB inhibits histone deacetylases, followed by FOXO activation. Thus, metabolites might affect aging or aging-related diseases by modulating signal modules [79,101,102,103,104]. As one hint of human aging research, results of whole blood metabolomics help to better understand biological age, especially given the variable nature of aging.

## Figures and Tables

**Table 1:** Fourteen metabolites for aging overlap with those for fasting and frailty.

Metabolites	Abundance	Levels in Elderly	Levels in Fasting	Levels in Frailty	Fraction	Role in Blood
1,5-anhydroglucitol (1,5-AG)	H-M	↓		↓	RBC	Antioxidant
acetyl-carnosine	L	↓		↓	Plasma	Antioxidant
carnosine	L	↓	↑		Plasma	Antioxidant
ophthalmic acid (OA)	H-M	↓	↑	↓	RBC	Antioxidant
leucine	H-M	↓	↑	↓	Plasma	Muscle maintenance
isoleucine	H-M	↓	↑	↓	Plasma	Muscle maintenance
NAD^+^	H-M	↓			RBC	Redox homeostasis
NADP^+^	H-M	↓			RBC	Redox homeostasis
UDP-acetyl-glucosamine	L	↓			RBC	Sugar nucleotide
citrulline	H-M	↑			Plasma	Urea cycle
pantothenate	H-M	↑	↑		RBC	Precursor of CoA
dimethyl-guanosine	L	↑			Plasma	Urine compound
*N*-acetyl-arginine	L	↑			Plasma	Urea cycle
N6-acetyl-lysine	L	↑			Plasma	Acetylated amino acid

Whole blood metabolomics reported 14 metabolites as aging markers. Of these, six metabolites were enriched in RBCs (red). The upper panel (blue box) shows nine metabolites that decrease in the elderly, while the lower panel (grey boxes) list metabolites that increase in the elderly. Notice that several compounds overlap with markers for fasting or frailty. Properties of metabolites are added. Metabolites with peak area > 10^7^ AU are shown as H-M (High to Medium) in red, and metabolites with peak area <10^7^ AU are shown as L (Low) in blue.
